# Partnering to Build Human Resources for Health Capacity in Africa: A Descriptive Review of the Global Health Service Partnership’s Innovative Model for Health Professional Education and Training From 2013-2018

**DOI:** 10.34172/ijhpm.2020.228

**Published:** 2020-11-29

**Authors:** Vanessa B. Kerry, Bonaventure Ahaisibwe, Bridget Malewezi, Deo Ngoma, Patricia Daoust, Eileen Stuart-Shor, Clelia Anna Mannino, Dick Day, Laura Foradori, Sadath A. Sayeed

**Affiliations:** ^1^Seed Global Health, Boston, MA, USA.; ^2^Massachusetts General Hospital, Boston, MA, USA.; ^3^Harvard Medical School, Boston, MA, USA.; ^4^Seed Global Health, Kampala, Uganda.; ^5^Seed Global Health, Lilongwe, Malawi.; ^6^ASCEND Program/Crown Agents, Dar es Salaam, Tanzania.; ^7^Center for Global Health, Massachusetts General Hospital, Boston, MA, USA.; ^8^University of Massachusetts Boston, Boston, MA, USA.; ^9^Beth Israel Deaconess Medical Center, Boston, MA, USA.; ^10^Catholic Medical Mission Board, New York City, NY, USA.; ^11^Former US Peace Corps, Washington, DC, USA.; ^12^Boston Children’s Hospital, Boston, MA, USA.

**Keywords:** Capacity Building, Africa, Medicine, Nursing, Midwifery, Health System Strengthening

## Abstract

Several Sustainable Development Goals (SDGs) (3, 16, 17) point to the need to systematically address massive shortages of human resources for health (HRH), build capacity and leverage partnerships to reduce the burden of global illness. Addressing these complex needs remain challenging, as simple increases in absolute numbers of healthcare providers trained is insufficient; substantial investment into long-term high-quality training programs is needed, as are incentives to retain qualified professionals within local systems of care delivery. We describe a novel HRH initiative, the Global Health Service Partnership (GHSP), involving collaboration between the US government (President’s Emergency Plan for AIDS Relief [PEPFAR], Peace Corps), 5 African countries, and a US-based non-profit, Seed Global Health. GHSP was formed to enlist US health professionals to assist in strengthening teaching and training capacity and focused on pre-and in-service medical and nursing education in Malawi, Tanzania, Uganda, Eswatini and Liberia. From 2013-2018, GHSP sent 186 US health professionals to 27 institutions in 5 countries, helping to train 16 280 unique trainees of all levels. Qualitative impacts included cultivating a supportive classroom learning environment, providing a pedagogical bridge to clinical service, and fostering a supportive clinical learning and practice environment through role modeling, mentorship and personalized learning at the bedside. GHSP represented a novel, multilateral, public-private collaboration to help address HRH needs in Africa. It offers a plausible, structured template for engagement and partnership in the field.

## Introduction

 Since 2015, the United Nations Sustainable Development Goals (SDGs), including 3, 16, and 17, have helped highlight the need to systematically address massive shortages of human resources for health (HRH), to build capacity of local institutions and to leverage partnership towards these goals, respectively, to reduce the burden of global illness; target 3.C specifically has called to “substantially increase health financing and the recruitment, development, training and retention of the health workforce in developing countries.”^1^ This acknowledges a reality first described by the UN Director General in the landmark 2006 World Health Organization (WHO) World Health Report: “People are a vital ingredient in the strengthening of health systems.”^2^ In the face of the coronavirus disease 2019 (COVID-19) global pandemic, the truth of this statement has never been more apparent.

 The challenge of how to effectively strengthen the global HRH sector remains daunting. In 2006, the World Health Report observed that a “solution is not straightforward, and there is no consensus on how to proceed.”^1^ This observation remains true in 2020. Critical shortages of multiple cadres of health workers exist in almost half the countries around the world.^3^ In 2015, a 7.2 million health professionals gap existed in 83 countries. Under current trends, this will grow to 18 million by 2035. As understanding of the global healthcare workforce crisis has deepened, so has appreciation of the tremendous socio-economic-political complexities frustrating its successful correction.

 Addressing this problem not only requires increases in the absolute numbers of healthcare providers trained, but also more investment in the quality of that training, and building meaningful incentive structures to retain qualified professionals within local systems of care delivery.^4-6^ There is growing interest in institutional-based, health professional capacity building programs.^7-9^ Many African academic institutions have increasingly sought partnerships to help create or improve training programs. The most ambitious of such endeavors has been the HRH Program in Rwanda, which set out to train over 7 years nearly all the health professionals and ancillary staffing required to run the country’s health system.^10^ Liberia was developing a similar program when it was interrupted by the massive Ebola outbreak in 2014. Post-Ebola reinvigorating this plan remains a top priority.^11^

 The Global Health Service Partnership (GHSP) was established in 2012 as a collaboration between the Peace Corps, the US President’s Emergency Plan for AIDS Relief (PEPFAR), and the non-profit, Seed Global Health (Seed), to help address the HRH crisis in sub-Saharan African countries with a high prevalence of HIV/AIDS. At that time, Africa bore 75% of the world’s burden of HIV, 25% of the global burden of disease, but had only 3% of the world’s healthcare workforce^2,12^; many countries lacked core faculty to help close these gaps.^13^ Aligned with several SDGs, GHSP’s goal was to help strengthen existing professional health education systems and care delivery by collaborating with partner countries to meet their immediate and long-term professional HRH needs. The model centered on sending US medical, nursing and/or midwifery health professionals to serve as teaching faculty in training institutions in resource-limited countries. This paper is the first descriptive accounting of the novel 5-year effort between 2013 and 2018.

## Methods

###  Program Creation and Description

 In 2011, Seed approached Peace Corps with a novel idea to create a government program to build healthcare capacity in countries of need. Peace Corps had already begun expanding professional volunteer placements with technical expertise through its Peace Corps Response Volunteer program. The idea behind GHSP also aligned with PEPFAR’s 2010-2014 strategic goal to increase the training of healthcare workers towards health system strengthening in countries of need.^14^ This convergence of interests led to a Peace Corp initiated request to PEPFAR for pilot funding to invest in an HRH strengthening program.

**Figure 1:**
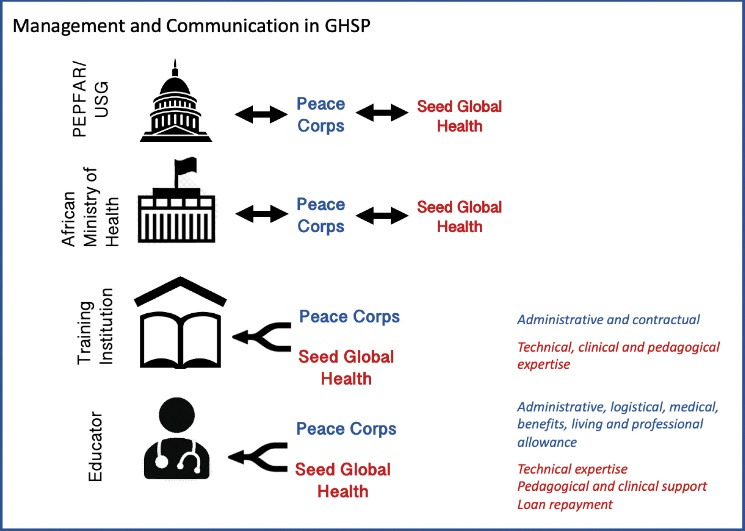
Management and Coordinating Relationships in GHSP. Abbreviations: GHSP, Global Health Service Partnership; PEPFAR, president’s Emergency Plan for AIDS Relief; USG, United States Government.

 GHSP was federally funded from 2013-2018. Eligible US citizen physicians, nurses, and midwives were recruited to serve as Peace Corps volunteers (“Educators”) within host African training institutions for one academic year. Educators received logistical and administrative support from Peace Corps as well as basic living and professional stipends that were aligned with local costs of living. Seed provided technical, pedagogical, and clinical support as well as recruitment, selection, and placement support of the Educators (See Figure 1). Seed also provided debt repayment stipends through independently raised funding to help offset the financial burden of participating in a volunteer-framed service program. Educators were eligible for up to $30 000 for each year determined by their level of proclaimed debt.

 A constellation of factors including the regional need, PEPFAR prioritization of countries to train health workers, language, and country-specific US mission interest in hosting GHSP led to the final selection of countries to launch the program. Operationally, GHSP approached potential host country governmental ministries to assess receptivity to the program. Upon agreement, GHSP first asked health and education ministers to identify specific training institutions, and then, leadership within those institutions refined pre-clinical and clinical teaching placements for a given year. Partner institutions included regional medical, nursing and midwifery schools and affiliated clinics and hospitals. An institution was defined as an educational entity that oversaw the training program of a cadre of health professionals. An institution could have a single site or multiple sites for placement. GHSP launched the first cohort of volunteer Educators in the countries of Malawi, Tanzania and Uganda. In 2016, at the request of PEPFAR, GHSP expanded to Liberia (post Ebola) and Eswatini (formerly Swaziland).

 Physician Educators were required to be board-eligible or board-certified. Nurse Educators were required to hold a current RN [registered nurse] license, have a Bachelor in Nursing degree, as well as a minimum of 3 years of clinical and/or teaching experience. Board certification in advanced practice nursing or a related field was preferred. Terms of reference and job responsibilities for each educator were established in close collaboration between the host country institutions, Seed and Peace Corps. GHSP in-country host partner sites reviewed the recommended candidates prior to final approval and placement. All Educators completed host country regulations for verification of credentials and licensure.

**Figure 2:**
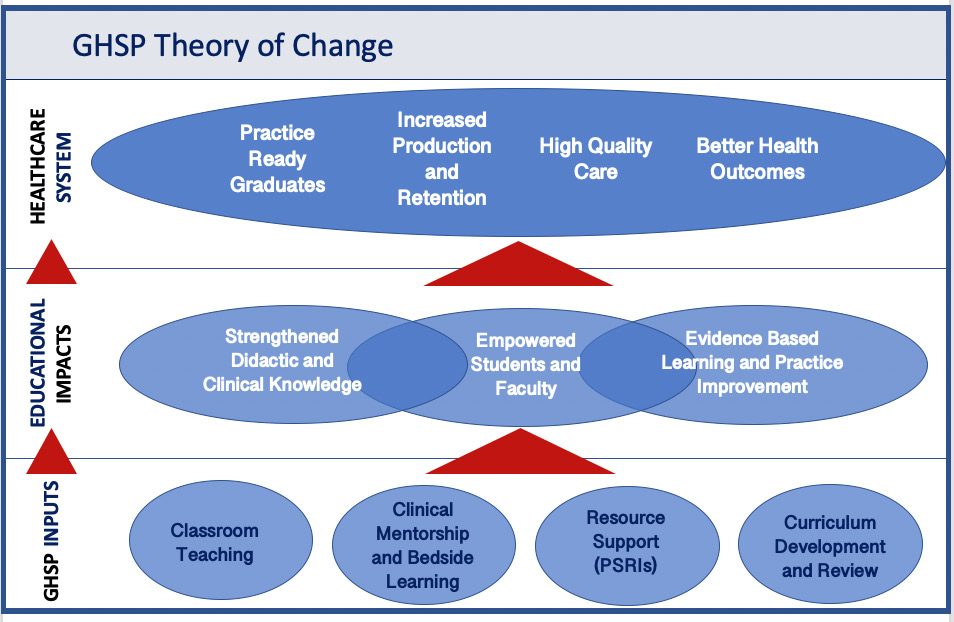
GHSP Theory of Change From Program Inputs to Long-term Impact. Abbreviation: GHSP, Global Health Service Partnership.

 As embedded pre-clinical and clinical faculty, GHSP Educators integrated into institutional roles, serving as lecturers, course directors, clinical mentors and supervisors. Educators worked alongside local faculty to enhance the quality and breadth of medical and nursing training and clinical practice. They taught in classroom and clinical settings. Provision of direct clinical care was primarily within the context of education to assist local students, trainees, faculty and staff, where the focus was on the delivery of quality by combining theoretical knowledge with clinical competency.^3^ GHSP theory of change is shown in Figure 2. All Educators underwent a multi-week comprehensive orientation both in the United States and in their host country and additional country service trainings occurred during the course of the year.

 Acknowledging the frequent lack of essential training resources, a grants program specific to GHSP was initiated Program Support Resources Initiative in year 3. Educators collaborating with their local colleagues could apply for one of 4 financial awards: micro-grants of up to $500 for items that supported an educational enterprise; practice improvement awards of up to $2000 that would support evidence-based, locally tailored improvement projects; equipment and training awards of up to $5500 to support equipment for skills labs, clinical assessment starter kits, or other investments where training on equipment could be incorporated into application; and, conference awards up to $7500 to support dissemination and generation of *in-country* science and promote networking, leadership, and local best practices.

###  Data Collection and Analysis

 GHSP’s monitoring, evaluation and learning efforts centered on 4 components – programming and operations, Educator activities and experiences, local student and faculty outcomes and experiences, and institutional outcomes and experiences. Both quantitative and qualitative data were collected to build an understanding of the program’s outputs and outcomes. Quantitative measures of productivity (activities, hours, number of trainees and deliverables) were collected monthly from Educators using validated, quantitative reporting forms. Regular check-ins with the Educators verified the accuracy of reporting. Qualitative measures of program outcomes were assessed through a series of internal and external evaluations conducted over the course of GHSP’s 5 years of implementation. These data collection efforts included feedback from GHSP’s key stakeholders – Educators, students, faculty colleagues, clinical colleagues, department leadership and institutional leadership. Analysis of each formal evaluation utilized a grounded theory approach. Evaluation findings from across the program’s 5 years of implementation were then subjected to an additional thematic synthesis to identify GHSP’s core outcome domains.

## Results

###  Quantitative Outputs

 GHSP partnered with 11 institutions in its first year at 14 sites in Malawi, Tanzania and Uganda. Over the subsequent 4 years, GHSP partnered with 16 additional institutions and 25 sites including expanding to Liberia and Eswatini. Over the course of the 5 year program, GHSP partnered: in Eswatini, with 4 institutions at 4 sites, in Liberia with 5 institutions at 8 total sites; in Malawi, with 6 institutions, at ten sites; in Tanzania with 7 institutions at ten sites, and in Uganda with 5 institutions at 7 sites (see Table 1). Six of the institutions across the 5 countries hosted both physicians and nurses or midwives. Sites are shown in Table 1.

**Table 1:** Global Health Service Partnership Countries, Institutions and Sites From 2013-2018

**Country**	**Institution**	**Site**	**GHSP Year**				
			**2013-2014**	**2014-2015**	**2015-2016**	**2016-2017**	**2017-2018**
**Liberia**	College of Medicine	COM-JFK Hospital				•	
	Liberia College of Physicians and Surgeons	CB Dunbar Hospital					•
		LCPS-JFK Hospital				•	•
		Phebe Hospital				•	•
		Redemption Hospital				•	•
	Mother Patern College of Health Sciences	Mother Patern College of Health Sciences				•	•
	Phebe Paramedical Training Centre	Phebe Paramedical Training Centre^a^				•	•
	Tubman National Institute of Medical Arts	Tubman National Institute of Medical Arts^a^				•	•
**Liberia total**	**5 Institutions**	**8 Sites**				**7**	**7**
**Malawi**	College of Medicine	COM-Blantyre	•	•	•	•	•
		COM-Lilongwe	•	•			•
		COM-Mangochi^a^		•	•	•	•
	Daeyang University	Daeyang University				•	•
	Kamuzu College of Nursing	KCN-Blantyre	•	•	•	•	•
		KCN-Lilongwe	•	•	•	•	
	Mzuzu University	Mzuzu University	•	•	•	•	•
	St. John of God College of Health Sciences	St. John of God College of Health Sciences^a^				•	•
	Umodzi Family Centre	Umodzi Family Centre MD					•
		Umodzi Family Centre RN					•
**Malawi total**	**6 Institutions**	**10 Sites**	**5**	**6**	**5**	**7**	**9**
**Eswatini**	Good Shepherd College of Nursing	Good Shepherd College of Nursing					•
	Southern Africa Nazarene University	Southern Africa Nazarene University^a^				•	•
	Swaziland Christian University^c^	Swaziland Christian University^c^					•
	University of Eswatini^b^	University of Eswatini^b,a^				•	•
**Eswatini total**	**4 Institutions**	**4 Sites**				**2**	**4**
**Tanzania**	Bugando Medical Center/CUHAS	Bugando Medical Center^a^	•	•	•	•	•
	Hubert Kairuki Memorial University	Hubert Kairuki Memorial University MD		•	•	•	•
		Hubert Kairuki Memorial University RN		•	•	•	•
	Mirembe School of Nursing	Mirembe School of Nursing	•	•			
	Muhimbili University of Health and Allied Sciences	Muhimbili University of Health and Allied Sciences MD	•	•	•	•	
		Muhimbili University of Health and Allied Sciences RN		•	•	•	•
	Mvumi Clinical Officer Training Centre	Mvumi Clinical Officer Training Centre	•	•		•	
	Sengerema Designated District Hospital	Sengerema Designated District Hospital	•	•	•	•	
	University of Dodoma	University of Dodoma MD		•		•	•
		University of Dodoma RN^a^		•	•	•	•
**Tanzania total**	**7 institutions**	**10 Sites**	**5**	**10**	**7**	**9**	**6**
**Uganda**	Busitema University	Busitema University MD			•	•	•
		Busitema University RN^a^				•	•
	Gulu University	Gulu University	•	•	•	•	
	Lira University	Lira University^a^	•	•	•	•	•
	Mbarara University of Science and Te chnology	Mbarara University of Science and Technology MD	•	•	•	•	•
		Mbarara University of Science and Technology RN	•	•	•	•	•
	Muni University	Muni University^a^			•	•	•
**Uganda total**	**5 Institutions**	**7 Sites**	**4**	**4**	**6**	**7**	**6**
**Grand Total**	**27 Institutions**	**39 Sites**	**14**	**20**	**18**	**32**	**32**

Abbreviations: GHSP, Global Health Service Partnership; CUHAS, Catholic University of Health and Allied Sciences; MD, medical doctor; RN, registered nurse. ^a^Indicates new program started with support of GHSP. ^b^Formerly known as University of Swaziland. ^c^Of note, Swazi Christian University lost their accreditation during the first year of our programming and the Educators were relocated to another site in Eswatini.

 Between July 2013 and September 2018, GHSP deployed 186 US professional Educators. The number of placements annually ranged from 31 to 69 (Table 2). Seventy-eight of the Educators were physicians, 89 were nurses and 19 were midwives. Data on age was available to Seed for the final 2 years of the program. In those 2 years, the average age was 45 for the physicians and 47 for the nurses and midwives with the youngest serving Educator aged 26 and the oldest aged 75.

**Table 2:** Total Number of Educators Annually by Country and Discipline From 2013-2018

**Country and Program**		**2013-2014**	**2014-2015**		**2015-2016**		**2016-2017**		**2017-2018**		**Total Unique Educators Placed**	**Total Extending Educators**	**Total Placements**
		**New**	**New**	**Extender**	**New**	**Extender**	**New**	**Extender**	**New**	**Extender**			
Liberia	RN						2	0	1	1	3	1	4
	Midwife						2	0	1	1	3	1	4
	MD						4	0	2	2	6	2	8
Malawi	RN	5	7	0	5	2	9	2	4	3	30	7	37
	Midwife	1	0	0	0	0	1	0	0	1	2	1	3
	MD	5	4	2	4	0	7	1	3	1	23	4	27
Tanzania	RN	3	6	1	4	1	5	0	2	3	20	5	25
	Midwife	1	2	0	2	1	3	1	1	0	9	2	11
	MD	5	7	0	3	1	7	1	1	3	23	5	28
Eswatini	RN						4	1	7	1	11	2	13
	Midwife						1	0	1	0	2	0	2
	MD						0	0	0	0	0	0	0
Uganda	RN	5	6	1	6	0	6	2	2	3	25	6	31
	Midwife	0	0	0	0	1	2	0	1	1	3	2	5
	MD	6	6	0	4	0	6	2	4	1	26	3	29
Total Educators		31	38	4	28	6	59	10	30	21	186	41	227
Total number of people serving each year		31	42		34		69		51				

Abbreviations: MD, medical doctor; RN, registered nurse.

 The majority of physicians serving had 5 or less years of post-training experience. The majority of nurses and midwives had more than 6 years of experience. Of the 78 physicians, 23 had additional degrees beyond their MD. Eighty-three of the 89 nurses that participated in the program had a graduate level degree in addition to their BSN. Sixty-five held Masters level degrees and 18 had earned their PhD. Of the 19 midwives, 15 held a Masters degree and 3 had PhDs.

 Forty-one individual Educators participated in the program for more than one year resulting in a total of 227 Educator placements over the 5-year program (see Table 2). While most Educators continued at their original site, 6 Educators extended their service in a different site and 5 Educators transitioned to other GHSP partner countries. Three Educators served in the program non-consecutively.

 GHSP assisted in the formal education of 16 280 unique trainees, 7315 of which were students, faculty or staff in medicine, and 8965 in the nursing or midwifery disciplines (see Table 3). While the majority of training for physicians was focused on medical students and residents/graduate students, clinical officers, physician assistants, and assistant medical officers were also taught. GHSP Educators may have taught the same individuals over successive years so that when counted in mentorship years defined as faculty-student interaction, GHSP provided 22 798 mentorship years (see Table 3). GHSP supported 11 new training programs or sites (10 nursing/midwifery and 1 medicine) and supplemented faculty at 28 existing (See Table 1).

**Table 3:** Number and Cadre of Trainees Taught by GHSP Educators Annually and in Total from 2013-2018

**Trainee Type**	**2013-2014**	**2014-2015**	**2015-2016**	**2016-2017**	**2017-2018**	**Unique Total**	**Cumulative Total** ^a^
**Medicine**							
Additional students outside department	33	28	20	24	84	189	189
Clinical officer/PA/AMO students	268	273	199	207	31	978	1102
Clinical staff	142	67	13	220	72	514	514
Faculty	0	13	3	37	38	91	91
Medical students	740	887	645	1367	371	4010	6614
Nursing students	0	0	0	522	438	960	960
Post graduates/MMed, interns and MSc	52	133	61	247	80	573	616
Medicine total	1235	1401	941	2624	1114	**7315**	**10086**
**Nursing**							
BSN	27	22	29	14	0	92	109
BSN midwifery	0	77	267	324	198	866	1425
BSN students	508	845	747	1836	793	4729	7703
Clinical staff	0	208	71	117	86	482	482
Diploma students	87	383	27	287	113	897	1079
Faculty	89	64	31	68	37	289	295
Medical/CO/PA students	0	0	0	229	65	294	199
Nursing post-graduate students	0	45	5	145	97	292	396
Other individuals	0	48	4	45	0	97	97
Other students	19	56	242	268	342	927	927
Nursing total	730	1748	1423	3333	1731	**8965**	**12712**
**Grand total**	**1965**	**3149**	**2364**	**5957**	**2845**	**16280**	**22798**

Abbreviations: GHSP, Global Health Service Partnership; BSN, Bachelor of Science in Nursing; PA, Physician’s Assistant; AMO, Assistant Medical Officer; CO, Clinical Officer. ^a^ Cumulative total reflects that individual trainees may have been taught over multiple years and are here counted as person-year-trained.

 GHSP Educators taught 851 courses over the 5-year period. The physician Educators taught 342 courses; nursing Educators taught 414 and midwifery Educators taught 95. Examples of courses and subjects taught included: didactic lectures on principles of medicine, nursing and midwifery, and on specialty topics such as HIV prevention, infection and management, and basic clinical skills; skills lab teaching on chest tube insertion and cardiac ultrasound; and clinical teaching on labor and delivery and in the intensive care unit. Educators also engaged in clinical mentorship and preceptorship, overseeing ward rounds, outpatient clinics and direct oversight of patient care during rotations. GHSP helped establish simulation labs in a number of the sites, providing a safe environment to encourage clinical competence, reflective practice and inter-professional collaboration. Additionally, Educators supported student or faculty initiatives to organize conferences or dedicated trainings for a wider professional audience. GHSP Educators cared for patients primarily in the context of a training opportunity – such as bedside teaching or teaching ward rounds; 76% of all patients were reported to have been seen in such a context. Over the course of GHSP, 153 awards were provided through the Program Support Resources Initiative to support educational enhancements such as trainings, workshops or resources.

###  Summary of Qualitative Results

**Figure 3:**
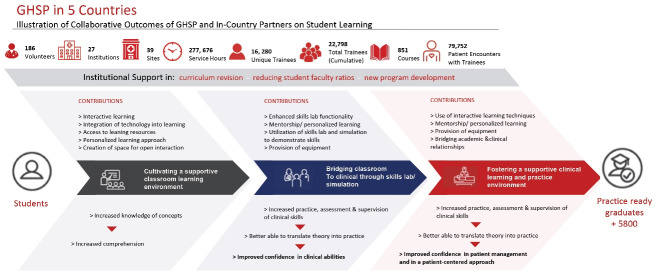
Qualitative and Collaborative Outcomes of GHSP on Trainee Learning. Abbreviation: GHSP, Global Health Service Partnership.

 We provide a very brief summary of GHSP’s qualitative impact here (a full description will be published in a separate manuscript). The program had 3 measured effects on the quality and type of education delivered at partner sites (see Figure 3). First, Educators helped cultivate a supportive classroom learning environment. They provided personalized learning approaches, encouraged open interaction and question asking, and nurtured interactive learning in the classroom. For example, Educators and faculty at Southern Africa Nazarene University in Eswatini used video recordings of students role-playing therapeutic communication and techniques to help instruct in the community mental health course. Through reviewing their recorded approaches, students engaged in their own learning and assessment. Educators also integrated technology into the learning and education process. As example, Educators and their counterparts at Hubert Kairuki Memorial University and University of Dodoma in Tanzania integrated Google Classroom in both medical and nursing instruction. Google Classroom provided a platform to streamline communication and expedite feedback between faculty and students.

 Second, GHSP provided a pedagogical bridge from classroom to clinical care delivery. Educators participated in the set-up of or teaching in skills labs and clinical simulations. The simulated setting provided an important means for students to practice and improve their clinical skills with a trained staff member present to mentor and supervise them. This exposure to clinical training in a controlled environment increased students’ ability to translate theory into practice and improved students’ confidence in their clinical abilities. For example, faculty at Mzuzu University in Malawi and an Educator developed a tracheostomy training scenario based on the needs identified by the Mzuzu Central Hospital. The simulation session was conducted at the hospital to educate learners in the recognition and management of a patient in respiratory distress due to an obstructed tracheostomy, and on how to perform routine tracheostomy care. The scenario, written by faculty, was also published in *Cureus*.^15^

 Third, Educators helped foster a supportive clinical learning and practice environment through role modeling, and by providing mentorship and personalized learning at the bedside. As with the simulation settings, the presence of Educators in the clinical setting allowed students to safely practice under guided supervision. Educators frequently extended the learning experience beyond the bedside by holding post-clinical conferences where trainees could discuss cases, positive and negative outcomes, the challenges of working in a resource limited setting, and how to provide compassionate, patient-centered care. GHSP provided some diagnostic equipment (for example, portable, handheld ultrasounds and iStats) which had the dual purpose of improving patient care and expanding tools for reinforcing anatomical and physiological principles in real time.

## Discussion

 The GHSP represented a unique human resource for health initiative that, during a 5-year span, augmented existing nursing and medical education in 5 African countries facing critical shortages of teaching faculty. Through multi-level (governmental and institutional) engagement with country partners, the program helped close curricular and training gaps in dozens of under-resourced health professional teaching institutions in each year of collaboration, and expanded local capacity for direct, supervised teaching of learners. The sheer number of individual trainees and courses taught over 5 years by 186 Educators evidences both the need for and impact of investing in health professional education in countries with critical faculty shortages. GHSP Educators did not replace or supplant local colleagues; instead, they reduced the teaching burden of an already vastly overstretched existing faculty within partnering institutions and enhanced their ability to better meet local training goals. Beyond quantifiable outputs, GHSP aimed to link experienced and enthusiastic US health professionals to eager and equally enthusiastic African student-learners and faculty colleagues.

 There are several limitations with the methods described in this report. A majority of the quantitative outputs presented derive from self-reported data collected by the Educators themselves. This may have introduced a potential bias and created an incentive for Educators to over or under report aspects of their work if they interpreted the reporting as an evaluation of their performance or as a reflection of limited time to devote to the evaluation activity. In addition, Educator reports may have been limited to what they could remember at the time of completion. Educators’ self-reported activities were not fully triangulated with data from host institution colleagues.

 Due to logistical and financial constraints, the program was unable to employ traditional research methodologies to measure potential impact across a range of activities. Assessing outcomes was challenging given the complexity of work undertaken across sites, and the program’s relatively small scale at numerous institutions (for example, only one Educator at a site in a given year). We report qualitative outcomes that are synthesized from both internal and external evaluations.

 Aggregated case studies and self-reported narratives support our observation that students experienced important professional growth because of their close association with a specific GHSP Educator; this corroborates previous findings that practicing nurses and physicians highly value mentoring experiences which are important to maturation and growth, in addition to developing responsibility and accountability as competent, compassionate care providers.^16,17^

 GHSP was not also organized to assess its impact on health outcomes of patients. Nevertheless, collected case studies and self-reported narratives revealed a consistently positive influence on patient care. For example, a Program Support Resources Initiative grant led to an evidence-based training on appropriate narcotic use on a surgical ward, which subsequently changed clinical practice. In a separate setting, one Medical Dean shared that Educators helped drop hospital mortality and improve retention of students within the system. The number of patients seen by Educators, their colleagues and trainees in the program was almost certainly underestimated.

 Working in the space of nursing, midwifery and medical education in systems severely constrained at multiple levels revealed numerous challenges and complexities. Even with the infusion of GHSP visiting faculty, adequate ratios for supervising trainees, especially at the clinical bedside, was infrequently achieved due to the remarkably high volume of patients in such settings and the persistent demands on existing faculty. We note that despite engaged and interested institutional leadership, a host of entrenched disincentives and structural barriers limited many efforts to produce sustainable improvements within many of these educational and trainee systems.

 Most faculty in resource limited settings juggle any number of demands that limit their available bandwidth for mentorship or clinical supervision. Faculty in many resource-constrained settings are mandated by university requirements, adopted (or inherited) from a Global North paradigm, to prioritize grant writing and research, oftentimes in a relationship with well-financed colleagues. It is important to recognize that while there is a need for locally relevant knowledge generation, this academic configuration predictably overburdens faculty with supervising a large number of students to do research (small sample, no controls, rarely published) that does very little to improve the health of their population, and pulls them away from the clinical training setting.

 Faculty are further disincentivized by historic barriers in health and education system design in African countries. Ministries of Health oversee and compensate the clinical workforce for their care in a hospital, health center or clinical setting. Ministries of Education are responsible for faculty teaching pedagogy of medicine and nursing in training institutions. Often, the result is that faculty are not paid to teach in the clinical setting, reducing availability of critical clinical mentorship and oversight. Because faculty committed to teaching are often inadequately compensated for classroom and clinical pedagogy, they are forced to redirect their limited professional energies to the more lucrative private sector.^13^

 Finally, there are constraints on hiring and retaining the workforce after training. Many Ministries of Health face difficult challenges in how to spend limited available funding. There are limited absolute funds with which to support multiple competing needs in the health system including infrastructure, health workforce, essential medicines and equipment, surveillance and data management. For example, Malawi has designated 11% of its 2017-2018 budget to support health but as the sixth poorest country in the world, in absolute dollars, this sum is only US$93 per person.^18^ Macroeconomic policies issued from international monetary bodies often control health sector spending. In Malawi, the Ministries of Health and Finance must balance hiring additional health workers in certain cadres with regulations set by the International Monetary Fund; these lenders strongly influence spending in a heavily externally-funded budget mode. As a result, there may be highly qualified, eager health professional graduates who cannot be hired.

 The multiple reasons that contribute to brain drain from the public sector have been well documented elsewhere^2,5,19^ but include not only insufficient salary levels but an inability to access continuing professional development, to have the tools to practice effectively in what one trains, and lack of career mobility or training opportunities.^2,19^ For example, in Cameroon, lack of opportunities or promotion, and desire to gain advanced training ranked above poor wages as reasons why healthcare professionals chose to migrate.^5^

 While most structural challenges cannot be fixed solely with programs like GHSP, it is critical for those committed to changing the global health landscape and achieving more equity to ask harder questions in this work, or risk continuing to pay lip service to lofty, yet needed, aspirations such as SDG 3. The default in many Global North health spheres remains to pursue narrow lines of activity and inquiry (disease, system, or technology-specific) that emphasize the agenda of funders and/or ourselves. Would-be Global North partners need to reflect genuinely with Global South partners on how to help assess and engage *the entire healthcare system*. Thoughtful and permanently impactful investments intended to improve the quality of health professional education can be made in these settings, but they must be done so as one part of a comprehensive approach at structural reform. To date, the HRH Rwanda program best exemplifies such a comprehensive approach; it is worth noting the degree of financial support that was required to fully operationalize that program.^10^ Against the backdrop of the current pandemic, the need for these investments is more evident than ever before.

## Conclusion

 Over the past decade, massive growth in the community of global health practitioners around the world has helped spur innovative efforts to better address the entrenched and profound healthcare disparities between affluent and poor. The GHSP model demonstrated that some health professionals are willing to forego or delay financial gain and/or accompanying professional prestige to make a tangible impact in the education of learners forced to train under conditions of extreme resource constraint. While the program was a modest effort in the scope of global need, it provided a template for engagement and partnership in the HRH field to build capacity and help leverage multi-stakeholder partnerships towards a common goal.

## Acknowledgements

 We would like to thank the following contributors to the GHSP including our collaborating partners in Uganda, Malawi, Tanzania, Eswatini and Liberia, the Educators, our Senior Advisors/Advisory Committee, PEPFAR, and Peace Corps. We would like to acknowledge the Seed Global Health staff including Julie Anathan, Irene Atuhairwe, Elizabeth Cunningham, Katelyn Fleming, Esther Johnston, Maria Lopez, and Aazamina Sud for data collection and support in preparation of the manuscript. We would like to also acknowledge Dr. Fitz Mullan for his extensive work in the HRH field and for his vision, support and energy which were critical to the development, launch, growth and impact of GHSP.

## Ethical issues

 This paper describes data and outputs that were reported, collected, and analyzed as part of an internal monitoring, learning, and evaluation process. In consultation with all partners to this project, it was determined that IRB approval was not ethically required, since the data collected was not part of a formal human subjects research study.

## Competing interests

 The authors all contributed to the development and oversight of GHSP.

## Authors’ contributions

 VBK, BA, BM, DN, PD, ESS, DD, LF, and SAS Substantial contributions to the conception and design of the work. VBK, BA, BM, DN, PD, ESS, CAM, DD, LF, and SAS were involved in acquisition, analysis and interpretation of data. VBK and SAS wrote the first draft of the article with substantial review and revision from all other authors.

## Disclaimer

 The analysis, conclusions and views expressed in the article are our own and do not reflect the position any partner institutions or funders (eg, Peace Corps/PEPFAR).

## Funding

 Support for the work documented in the manuscript was provided by the Presidents Emergency Plan for AIDS Relief (PEPFAR), the Abbot Fund, Bank of America Foundation, Bohemian Foundation, Child Relief International, Draper Richards Kaplan, Engelhard Foundation, ExxonMobil Foundation, FedEx, Gradian Health Systems, Medtronic/ Covidien Foundation, Pfizer Foundation, Schooner Foundation, and Wyss Medical Foundation.
